# Vasovagal Syncope With Sinus Arrest Triggered by Venipuncture in a Patient Receiving Multiple Psychotropic Medications: A Case Report

**DOI:** 10.7759/cureus.98991

**Published:** 2025-12-11

**Authors:** Aki Kawauchi, Ayumi Nakayama, Yushi Abe, Yoko Yamazaki, Shigeru Maeda

**Affiliations:** 1 Department of Dental Anesthesiology, Graduate School of Medical and Dental Sciences, Institute of Science Tokyo, Tokyo, JPN

**Keywords:** bispectral index, general anesthesia, psychotropic medications, sinus arrest, vasovagal syncope

## Abstract

Vasovagal syncope (VVS) is commonly described as the most frequent form of reflex syncope and is often regarded as benign. However, in some cases, VVS may progress to profound circulatory collapse with sinus arrest or even cardiac arrest, necessitating resuscitation. Previous reports have indicated that the concomitant use of multiple psychotropic medications, particularly antipsychotic agents, may influence autonomic regulation and hemodynamic stability, potentially lowering the threshold for VVS. As a result, even minor procedural stimuli can precipitate severe VVS in susceptible individuals.

A 29-year-old man with a history of anxiety, depression, and insomnia was receiving duloxetine, quetiapine, and several hypnotic agents and was scheduled for third molar extraction under general anesthesia. During intravenous cannulation, progressive bradycardia culminated in sinus arrest. Chest compressions and administration of atropine restored spontaneous circulation and consciousness approximately 45 seconds later, although bradycardia persisted until hemodynamic stability was achieved following ephedrine administration. Notably, bispectral index (BIS) values declined before the onset of bradycardia and hypotension, suggesting early central nervous system involvement in the syncopal cascade.

This case highlights that in patients receiving multiple psychotropic agents, even minor procedures can precipitate severe VVS. Psychotropic medications may lower the syncope threshold, and BIS monitoring may help identify early changes indicative of impending events.

## Introduction

Vasovagal syncope (VVS), the most prevalent reflex syncope, is defined as a transient loss of consciousness secondary to bradycardia, vasodilation, and hypotension [1-3]. VVS may be triggered by pain, procedures, upright posture, or emotional stress and is often accompanied by prodromal symptoms [3]. Even young individuals without comorbidities can develop marked bradycardia or transient cardiac arrest, occasionally necessitating resuscitative interventions [4]. In the perioperative setting, VVS is strongly influenced by the convergence of surgical stress, pain, dehydration, anxiety, positional changes, and anesthetic agents [5]. Therefore, vigilant monitoring and prompt intervention are essential [1].

Although the pathophysiology of VVS remains incompletely understood, the Bezold-Jarisch reflex is considered a principal mechanism, whereby enhanced parasympathetic tone and reduced sympathetic activity cause bradycardia, vasodilation, and syncope [2]. Recent studies suggest that structural and functional alterations within the central autonomic network may contribute to individual susceptibility to syncope [6,7].

Pharmacological factors also play a role. Antipsychotics and antidepressants may induce vasodilation and impair circulatory regulation [8,9], while hypnotics can reduce blood pressure responses through central nervous system depression and decreased venous return [10,11]. Although each agent alone increases syncope risk, concomitant use may further enhance vulnerability to VVS.

VVS can precipitate profound circulatory collapse, even after minor stimuli; therefore, in the perioperative setting, preparedness for unpredictable events is essential. Herein, we report a case where intravenous cannulation prior to induction of general anesthesia triggered severe VVS with sinus arrest and generalized tonic seizures. A decline in the bispectral index (BIS) preceded bradycardia and hypotension, suggesting early central nervous system involvement. Furthermore, the patient was on multiple psychotropic medications, raising the possibility that pharmacological factors lowered the threshold for syncope.

This report underscores the importance of vigilance in perioperative VVS management and provides clinical insights into VVS occurrence and BIS alterations in patients receiving psychotropic therapy. Written informed consent was obtained from the patient for the publication of this case report.

## Case presentation

The patient was a 29-year-old man (height: 178 cm; weight: 58 kg) diagnosed with bilateral horizontally impacted mandibular third molars and pericoronitis of the bilateral maxillary third molars. Because extraction under local anesthesia was unfeasible due to severe dental phobia, he was referred to our Department of Oral and Maxillofacial Surgery for extraction of the third molars under general anesthesia. His medical history included anxiety, depression, and insomnia managed with psychiatric care and pharmacotherapy. His regular medications included duloxetine 20 mg in the morning, quetiapine 12.5 mg at bedtime, and the hypnotics etizolam 1 mg, eszopiclone 1 mg, and trazodone 25 mg, all taken at bedtime. He was also prescribed a probiotic and a gastrointestinal prokinetic agent. He had no history of cardiac disease, surgery, or food or drug allergies. Airway evaluation was normal.

Preoperative blood tests, electrocardiography, and chest radiography performed three weeks before surgery revealed no abnormalities. He had no history of smoking and only consumed alcohol occasionally. His anesthetic risk was classified as American Society of Anesthesiologists (ASA) physical status II. Since his mid-teens, he had experienced recurrent vasovagal reflexes (VVR) during blood sampling and medical procedures, though never with loss of consciousness. Notably, during preoperative orientation three weeks before surgery, he reported presyncopal discomfort with transient hypotension (74/32 mmHg), bradycardia (52 bpm), and oxygen saturation of 98%.

The patient was admitted the day before surgery, fasted from midnight, and abstained from fluids from 7:00 am on the day of surgery. He consumed 200 mL of water up to two hours before entering the operating room, and no dehydration was observed on admission. Intravenous fluid was not administered preoperatively. On arrival in the operating room, non-invasive blood pressure monitoring, electrocardiography, pulse oximetry, and BIS monitoring were initiated. Supplemental oxygen was delivered via face mask at 6 L/min. His vital signs were stable: blood pressure 111/63 mmHg (mean arterial pressure, 79 mmHg), heart rate 79 bpm, and SpO₂ 100%. A 22-gauge IV catheter was inserted into the left forearm without premedication or topical anesthesia. Immediately after cannulation, his vital signs remained stable (blood pressure 112/60 mmHg, heart rate 60 bpm, SpO₂ 100%). During fixation of the intravenous line, his heart rate progressively declined, culminating in sinus arrest approximately 45 seconds after the onset of bradycardia. Immediately following the arrest, two generalized tonic seizures lasting 3-5 seconds occurred, with systolic blood pressure transiently decreasing to 76 mmHg. Oxygen saturation was maintained at 100% throughout the event. Immediately after confirming sinus arrest, chest compressions were initiated, and atropine (0.5 mg) was administered. Spontaneous cardiac activity resumed, and consciousness returned approximately 45 seconds after the onset of arrest. Persistent bradycardia of 30-40 bpm required 5 mg of ephedrine, leading to hemodynamic stabilization.

With oxygen administration, BIS values fluctuated between the 60s and high 80s. During this period, the patient was asked about any discomfort but denied symptoms. However, after leaving the operating room, he reported lightheadedness beginning with mask application (oxygen initiation). During intravenous cannulation, BIS decreased from 90 to the 70s, preceding bradycardia, and further declined to the low 50s at sinus arrest. Following restoration of circulation, BIS promptly returned to the 90s. Although sinus rhythm resumed and consciousness was restored, bradycardia persisted, during which BIS fluctuated between the 60s and high 80s. After ephedrine administration, both hemodynamic parameters and BIS stabilized around 90. The patient was then transferred to the emergency room for further evaluation and in anticipation of potential recurrence.

**Figure 1:**
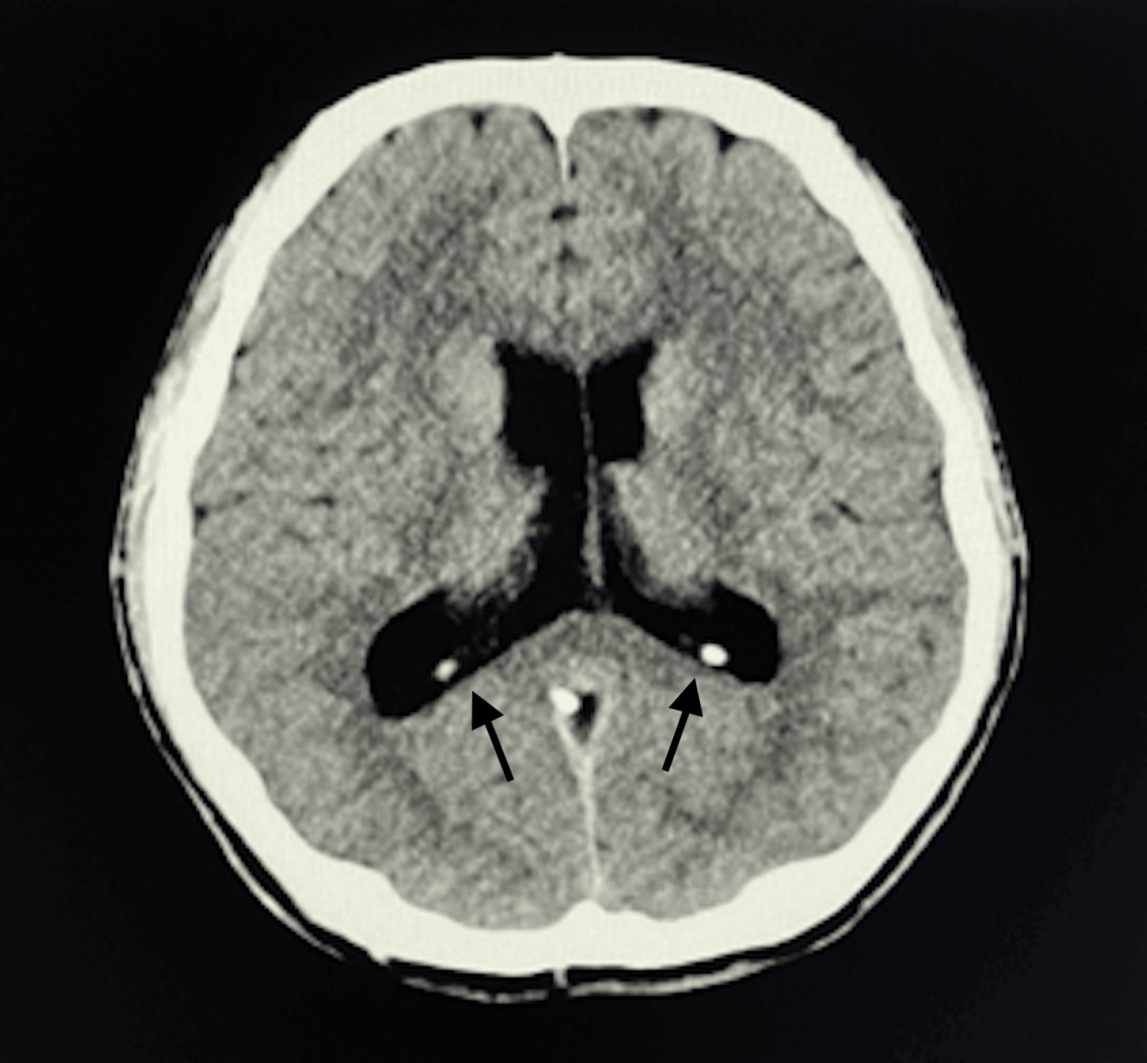
Brain computed tomography. Axial CT demonstrating marked enlargement of the posterior bodies and trigones of the bilateral lateral ventricles (arrows).

On arrival at the emergency room, his vital signs were stable, with a blood pressure of 94/60 mmHg, heart rate of 76 bpm, and oxygen saturation of 99% in room air; no subjective symptoms were reported. Laboratory test results were unremarkable except for mildly elevated creatine kinase (303 U/L; preoperative value 172 U/L; normal range: 50-240 U/L). Electrocardiography showed a normal sinus rhythm without ST-T changes. Head-to-pelvis computed tomography revealed marked enlargement of the posterior bodies and trigones of the bilateral lateral ventricles, suggestive of periventricular leukomalacia (Figure 1). Neurologists advised follow-up only, noting the absence of prematurity or low birth weight and limited clinical significance. The patient had no such history. After further evaluation in the emergency room, the patient was returned to the general ward approximately 2.5 hours after VVS onset. At the time of return, he was fully alert with stable vitals. The intravenous catheter was removed six hours after the syncopal event, at which time his vital signs remained stable (blood pressure 96/56 mmHg, heart rate 80 bpm, SpO₂ 98%, respiratory rate 13/min), and he remained asymptomatic. The patient was continuously monitored for blood pressure, SpO₂, and electrocardiography, and his subsequent clinical course remained uneventful. He was discharged the following day.

The patient remained asymptomatic from discharge through the one-week outpatient follow-up. Two weeks after discharge, he was re-evaluated by both neurologists and cardiologists. Although an electroencephalogram was not performed, the clinical course indicated that the seizures were attributable to cerebral hypoperfusion secondary to sinus arrest. Epilepsy was considered unlikely, and the event was ultimately concluded to be consistent with VVS. The cardiology team determined that the episode represented a cardioinhibitory type of VVS. They recommended that, if general anesthesia is required in the future, intravenous access and fluid administration be established preoperatively in the ward to prevent dehydration, with preparation for temporary pacemaker (tPM) insertion to manage potential cardioinhibitory responses. Continued follow-up in both neurology and cardiology clinics is planned to assess the appropriateness of future procedures under general anesthesia.

## Discussion

This case report describes severe VVS with sinus arrest and generalized tonic seizures triggered by a seemingly minor stimulus, intravenous cannulation. Even young patients without significant comorbidities can develop profound circulatory inhibition in response to pain or emotional stress [1,3,4]. International guidelines also emphasize that reflex syncope, although usually benign, may present with marked cardioinhibitory responses, including cardiac arrest, necessitating immediate resuscitation [3]. Although sinus arrest from a minor intervention such as intravenous access prior to induction of general anesthesia is rare, this case demonstrates how multiple contributing factors may converge to produce such a critical event.

A key consideration in this case was the concomitant use of multiple psychotropic agents, including antipsychotics, antidepressants, and hypnotics. Orthostatic hypotension is the most common autonomic adverse effect of antipsychotics and may cause dizziness, syncope, and falls [8,12]. Quetiapine carries a particularly high risk of orthostatic hypotension [8,12]. While the primary pharmacological actions of quetiapine involve serotonin 5-HT₂ and dopamine D₂ receptor antagonism, the drug also affects histamine H₁ and α₁-adrenergic receptors. The latter, α₁-adrenergic receptor blockade, induces peripheral vasodilation and may contribute to hemodynamic instability [8,13]. Furthermore, long-term antipsychotic use has been associated with accelerated gray matter loss, underscoring that the neural effects of these agents are complex and depend on drug class, treatment duration, and underlying psychiatric conditions [14].

Serotonin-norepinephrine reuptake inhibitors (SNRIs) alleviate depressive symptoms by inhibiting serotonin and norepinephrine reuptake in the synaptic cleft; however, their effects extend to the peripheral autonomic nervous system [9]. Augmented noradrenergic transmission regulates vascular tone and sympathetic responses but may also provoke blood pressure fluctuations, thereby increasing the risk of orthostatic hypotension and syncope [9]. Duloxetine, prescribed in this case, exerts such pharmacological effects and may have altered the circulatory response to venipuncture, thereby lowering the threshold for VVS. Hypotension and hemodynamic instability associated with SNRIs are well documented, suggesting these mechanisms contributed to the profound cardioinhibitory response observed. Previous studies similarly report associations between antidepressant use and orthostatic hypotension or syncope [9], underscoring the relevance of this pharmacological background in understanding the pathophysiology of the present case.

In addition, regular use of benzodiazepines and non-benzodiazepine hypnotics likely increased vulnerability. These agents enhance central inhibition through GABA-A receptor activity, reducing sympathetic outflow and diminishing venous return due to muscle relaxation [10]. Such mechanisms impair orthostatic blood pressure responses and are linked with drug-induced orthostatic hypotension, bradycardia, syncope, and increased risk of falls [11]. Furthermore, trazodone, widely prescribed as a sleep aid, exerts not only serotonin reuptake inhibition but also potent α₁-adrenergic receptor blockade. Several clinical studies demonstrate that even at low doses, trazodone is associated with orthostatic hypotension, syncope, and falls [15]. At the cellular level, trazodone inhibits multiple cardiac ion channels, including hERG (IKr), IKs, INa, and ICa, in a concentration-dependent manner, thereby prolonging action potential duration and inducing early after-depolarizations, which may precipitate QT prolongation and ventricular arrhythmias [16].

Collectively, concomitant use of these medications in the present case may have exerted synergistic effects on autonomic and cardiovascular regulation, thereby lowering the VVS threshold and increasing perioperative syncope risk. Beyond pharmacological influences, psychiatric symptoms may also heighten susceptibility. Bhangu JS et al. reported that in older adults, depressive symptom severity was significantly associated with a higher incidence of syncope, and tricyclic antidepressant use, in particular, increased syncope frequency [17]. In this patient, psychiatric symptoms and medication-related factors likely interacted to exacerbate the patient’s vulnerability to VVS.

Subsequent evaluation following the VVS episode incidentally revealed enlargement of the posterior bodies and trigones of the bilateral lateral ventricles. Although a direct causal relationship between these ventricular structural changes and the occurrence of VVS remains unclear, accumulating evidence suggests that individual variations in brain structure may influence syncope susceptibility. Kim JB et al. reported atrophy and reduced volume of the right insular cortex in patients with neurocardiogenic syncope [6], while Park BS et al. demonstrated reorganization of hub structures within the central autonomic network in patients with reflex syncope using graph-theoretical analysis [7]. Furthermore, Huh H et al. observed increased blood-brain barrier permeability following a single vasovagal reflex episode, suggesting transient cerebral microcirculatory alterations [18]. Kruit MC et al. reported significantly increased deep and periventricular white matter lesions in patients with recurrent syncope or orthostatic intolerance, suggesting possible circulatory dysregulation or impaired cerebral perfusion [19]. Collectively, these studies highlight structural and network-related brain abnormalities in patients with reflex syncope, although their connection to the ventricular enlargement observed in this case remains uncertain.

In this patient, BIS values declined before the onset of bradycardia and hypotension. This aligns with findings by Win NN et al., who reported an early BIS decrease during a vasovagal reflex episode [20]. In their study, BIS values dropped abruptly from 98 to 35 at the time of, or preceding, bradycardia and hypotension, coinciding with loss of consciousness. Although traditionally employed to monitor anesthetic depth, BIS may also detect early cortical activity suppression and alterations in consciousness during VVR. These findings suggest that BIS monitoring may help predict syncope events and guide timely interventions. However, prospective studies are warranted to establish the clinical utility of BIS in syncope monitoring.

The following considerations outline the major clinical implications derived from this case. A long-standing history of recurrent vasovagal reactions during medical procedures should be regarded as a perioperative red flag and explicitly incorporated into anesthetic planning. Careful evaluation and adjustment of psychotropic medications that impair autonomic regulation and hemodynamic stability is an essential clinical consideration in this case. These agents may substantially lower the threshold for vasovagal reactions and should be systematically reviewed during perioperative planning. The 2017 ACC/AHA/HRS syncope guidelines recommend that drug-induced orthostatic hypotension be actively considered during medication review or discontinuation (Class IIa). This underscores that pharmacologically induced blood pressure reduction is a significant risk factor for syncope and that adjustment of pharmacotherapy may be an effective preventive measure in perioperative management. Furthermore, the guidelines emphasize the need for comprehensive perioperative strategies developed through multidisciplinary collaboration.

Importantly, avoiding perioperative dehydration is of substantial preventive significance, particularly in patients with heightened susceptibility to vasovagal reactions. Establishing intravenous access in a calm, monitored area outside the operating room, where emergency treatment is immediately available, may further reduce the risk of VVS. An anticholinergic agent, such as atropine or glycopyrrolate, should be immediately available in the monitored setting for such patients. Additionally, in patients receiving psychotropic medications with known cardiovascular effects, as in this case, a decline in BIS values should be regarded as a potential early marker of severe vasovagal reflex episodes.

## Conclusions

This case report describes a severe episode of VVS with sinus arrest and generalized tonic seizures triggered by intravenous cannulation before induction of general anesthesia. The patient was receiving multiple psychotropic agents, including an SNRI, an antipsychotic, and both benzodiazepine and non-benzodiazepine hypnotics, whose pharmacological properties may impair autonomic regulation and hemodynamic stability, thereby lowering the syncope threshold. Notably, BIS monitoring revealed a decline in cortical activity preceding bradycardia and hypotension, suggesting possible involvement of CNS mechanisms. These observations indicate that BIS may serve as an early marker for impending VVS, although its clinical utility warrants further investigation.

This case emphasizes the perioperative risk of VVS in patients receiving psychotropic medications. In such patients, comprehensive perioperative strategies, including maintenance of hydration, reduction of procedural stress, and preparation for cardioinhibitory events, should be systematically considered.
